# Viral dysbiosis in children with new-onset celiac disease

**DOI:** 10.1371/journal.pone.0262108

**Published:** 2022-01-14

**Authors:** Mohammad El Mouzan, Asaad Assiri, Ahmed Al Sarkhy, Mona Alasmi, Anjum Saeed, Abdulrahman Al-Hussaini, Badr AlSaleem, Mohammad Al Mofarreh

**Affiliations:** 1 Department of Pediatrics (Gastroenterology), King Saud University, Riyadh, Kingdom of Saudi Arabia; 2 Department of Pediatrics, King Saud University Medical City, King Saud University, Riyadh, Kingdom of Saudi Arabia; 3 Department of Pediatrics, Prince Abdullah Bin Khalid Celiac Disease Research Chair, King Saud University, Riyadh, Kingdom of Saudi Arabia; 4 Division of Pediatric Gastroenterology, Children’s Specialist Hospital, King Fahad Medical City, Riyadh, Kingdom of Saudi Arabia; 5 Faculty of Medicine, AlFaisal University, Riyadh, Kingdom of Saudi Arabia; 6 Division of Gastroenterology, The Children Hospital, King Fahad Medical City, Pediatric Intestinal Failure and Parenteral Nutrition Program, Riyadh, Kingdom of Saudi Arabia; 7 Department of Gastroenterology, Al Mofarreh PolyClinic, Riyadh, Kingdom of Saudi Arabia; Arizona State University, UNITED STATES

## Abstract

Viruses are common components of the intestinal microbiome, modulating host bacterial metabolism and interacting with the immune system, with a possible role in the pathogenesis of immune-mediated diseases such as celiac disease (CeD). The objective of this study was to characterize the virome profile in children with new-onset CeD. We used metagenomic analysis of viral DNA in mucosal and fecal samples from children with CeD and controls and performed sequencing using the Nextera XT library preparation kit. Abundance log2 fold changes were calculated using differential expression and linear discriminant effect size. Shannon alpha and Bray–Curtis beta diversity were determined. A total of 40 children with CeD and 39 controls were included. We found viral dysbiosis in both fecal and mucosal samples. Examples of significantly more abundant species in fecal samples of children with CeD included *Human polyomavirus 2*, *Enterobacteria phage mEpX1*, and *Enterobacteria phage mEpX2*; whereas less abundant species included *Lactococcus phages ul36 and Streptococcus phage Abc2*. In mucosal samples however, no species were significantly associated with CeD. Shannon alpha diversity was not significantly different between CeD and non-CeD groups and Bray–Curtis beta diversity showed no significant separation between CeD and non-CeD samples in either mucosal or stool samples, whereas separation was clear in all samples. We identified significant viral dysbiosis in children with CeD, suggesting a potential role in the pathogenesis of CeD indicating the need for further studies.

## Introduction

Viruses are common components of the intestinal microbiome, among which, bacteriophages (viruses that infect bacteria) are the most common [1, 2]. Bacteriophages modulate host bacterial metabolism and interact with the immune system, triggering the innate and adaptive immune systems by employing similar mechanisms as those used by bacteria, suggesting a role in chronic inflammatory conditions [3, 4]. Animal studies in mice have suggested a possible protective role of noroviruses in inflammatory bowel disease [5, 6]. In humans, the association between intestinal viruses and inflammatory bowel disease (IBD) has been reported as well, characterizing viral dysbiosis in both Crohn’s disease and ulcerative colitis [7–10].

Celiac disease (CeD) is an autoimmune enteropathy with pathologic similarity to IBD. In genetically susceptible individuals, exposure to gluten results in inflammatory cascade, leading to chronic intestinal injury [11–13]. However, gluten exposure alone does not trigger CeD in all genetically susceptible individuals, suggesting a role of additional factors. In this context, microbiota have been reported as one of the most important environmental factors in CeD. Bacterial dysbiosis in patients with CeD demonstrated decreased abundance of “beneficial bacteria” such as *Bifidobacteria*, *Clostridia*, and *Lactobacilli* and enrichment of potentially pathogenic bacteria such as *Escherichia coli* and *Bacteroides* [14–16]. Similarly, fungal dysbiosis, especially *Saccharomyces* and *Candida*, has been described in children with CeD [17–20] However, less is known at this time regarding the role of viruses within the CeD population. Major classes of human gut virome include bacteriophages, DNA eukaryotic viruses, and RNA eukaryotic viruses [21]. It has been suggested that infection with RNA and possibly DNA eukaryotic viruses may cause a transient disease resulting in loss of tolerance to gluten and development of CeD in susceptible individuals [22]. Bacteriophages, on the other hand, could trigger CeD directly or by modifying bacterial dysbiosis associated with CeD. In this study we used shotgun metagenomic DNA sequencing with the objective to characterize the profile of bacteriophages and DNA eukaryotic viruses in a cohort of children with newly diagnosed CeD. Accordingly, RNA eukaryotic viruses were not analyzed.

## Patients and methods

### Study population

Children were enrolled from King Khalid University Hospital, King Saud University; King Fahad Medical City Children Hospital, Ministry of Health, and Al Mofarreh Polyclinics, a private medical institution, all of which are in Riyadh, the Kingdom of Saudi Arabia (KSA). The children attending the Gastroenterology Clinics were evaluated. Inclusion criteria in this study included age below 18 years, normal gluten containing diet, and no antibiotic exposure for at least 6 months. The study was introduced to the parents and children by one of the investigators, including explanation of all items in the informed consent form approved by the IRB. After signing the written consent form by one of the parents, the children were enrolled in the study. After complete investigations, the children with confirmed diagnosis of CeD were designated as cases and those in whom the diagnosis of CeD was excluded were designated as controls. Controls include healthy school children who gave stool samples. In total, there were two groups of children; the first group included those confirmed CeD who provided stool as well as mucosal samples (n = 20) or mucosal samples (n = 20). In this group, the diagnosis of CeD was confirmed according to the European Society of Pediatric Gastroenterology Hepatology and Nutrition guidelines [23]. The second group involved controls who gave stool samples, included a subgroup of 20 healthy schoolchildren who had negative anti–tissue transglutaminase-A (TTG-A) antibodies with normal serum immunoglobulin A levels taken from a larger random sample recruited for a mass screening study [24]. The other subgroup of non-CeD controls included 19 children who provided mucosal samples; these children presented with various symptoms but were TTG-A–negative with normal serum immunoglobulin A levels, normal endoscopy, and histopathology of the duodenal mucosa.

### Sample collection, storage, and retrieval

Mucosal samples from 20 children with confirmed untreated CeD and 19 non-CD controls were collected from D2 in cryovials without fixative or stabilizer and transported in ice to the central laboratory. Similarly, fecal samples were collected in cryovials from 20 children with CeD and 20 healthy controls and transported in ice to the same. All samples were initially stored in a freezer at −80°C; then, at the time of analysis were retrieved and dispatched by express mail in a temperature-controlled container filled with dry ice until delivery to the laboratory for metagenomic analysis (CosmosID, Rockville, MD, USA).

### DNA extraction and sequencing

DNA was isolated from mucosa samples using the Zymobiomics miniprep kit (Zymo Research, Irvine, CA, USA) and from stool samples using the DNeasy PowerSoil DNA kit (Qiagen, Hilden, Germany), with each process done according to the manufacturer’s instructions. Isolated DNA was quantified by Qubit (Thermo Fisher Scientific, Waltham, MA, USA).

DNA libraries were prepared using the Illumina Nextera XT library preparation kit, according to the manufacturer’s protocol. Library quantity and quality were assessed with Qubit and Tapestation (Agilent Technologies, Santa, Clara, CA, USA). Libraries were then sequenced on an HiSeq platform (2 × 150 bp; Illumina, San Diego, CA, USA).

### Bioinformatic and statistical analyses

Unassembled sequencing reads were directly analyzed with the CosmosID bioinformatics platform (CosmosID Inc., Rockville, MD, USA) described elsewhere for multi-kingdom microbiome analysis and quantification of each organism’s relative abundance [25–28]. Briefly, the system uses curated genome databases and a high-performance data-mining algorithm that rapidly disambiguates hundreds of millions of metagenomic sequence reads into the discrete microorganisms engendering the particular sequences.

#### Abundance analysis

*DESeq2 differential abundance analysis*. Differential abundance analyses were generated starting with the abundance score matrices from the CosmosID taxonomic analysis. Differential abundance values for organisms were calculated using DESeq2 from the Phyloseq package for R (R Foundation for Statistical Computing, Vienna, Austria). For the mucosal and stool samples separately, the log2 fold change and associated P-values for CeD vs non-CeD are displayed [29, 30].

*Linear discriminant analysis (LDA) of effect size (LEfSe)*. LEfSe figures were generated using the Galaxy web application, based on relative abundance tables from CosmosID taxonomic analysis, and calculated with a Kruskal–Wallis alpha value of 0.05, a Wilcoxon alpha value of 0.05, and a logarithmic LDA score threshold of 2.0 [31].

#### Breadth and depth analysis

The breadth and depth of coverage of across the genome for each of the 39 genomes by each of the four cohorts (mucosa celiac, mucosa non-celiac, stool celiac, stool non-celiac). For virus species with significant differences identified, the genomes were downloaded via NCBI RefSeq for mapping. All metagenomic samples were randomly subsampled to 9 million total reads using the reformat.sh script within the bbtools suite.

All subsampled metagenomic files were mapped against each of the 39 references using bowtie2 (parameters: bowtie2 -D 20 -R 3 -N 1 -L 25 -i S,1,0.50 -f -t -x). Number of total bases in the query sample and number of bases covered against the reference sample were collected from the output bam files using samtools. Coverage breadth was calculated as reference bases covered divided by reference length. Coverage depth average was calculated as total query bases mapped divided by reference length. Cohort average values for each reference have been provided.

#### Diversity analysis

*Alpha diversity*. Alpha diversity boxplots were calculated from the species-level abundance-score matrices from the CosmosID taxonomic analysis. Shannon alpha diversity metrics were calculated using the Vegan package for R. Also, t-tests were performed between each the CeD and non-CeD groups using the ggsignif package for R. Finally, boxplots with overlaid significance in p-value format were generated using the ggplot2 package for R [32–34].

*Beta diversity*. Beta diversity two-dimensional principal coordinate analyses (PCoAs) were calculated from the species-level relative-abundance matrices from the CosmosID taxonomic analysis. Bray–Curtis diversity was calculated using the Vegan package for R with the vegdist function and PCoA tables were generated using the Vegan package’s PCoA function. Plots were visualized using the ggpubr package for R [32, 35].

For all analyses in this report, P- value were corrected for false discovery rate and the difference was considered significant when the P-value was < 0.05.

### Ethical approval

This study was approved by the Institutional Review Board of the College of Medicine, King Saud University in Riyadh, Kingdom of Saudi Arabia (no. 14/4464/IRB). All parents gave written informed consent and since all children included in this study were below 18 years, one of the parents signed the written consent in their behalf.

## Results

### The study population

The demographic and clinical characteristics of the study population of 40 children with CeD and 39 controls are presented in Table 1. Males accounted for 28% and 41% of the children with CeD and the control group, respectively, and the median ages at diagnosis were 10.3, 11.3 and 10.6 years for children with CeD, controls fecal and mucosal samples, respectively. The number of asymptomatic children with CeD was 15 of 40 (38%), while the remainder (n = 25) showed various combinations of symptoms including anemia, growth impairment, and abdominal pain. Stool samples were given by 20 healthy controls, whereas all 19 mucosal samples were provided by children with various symptoms, with functional abdominal pain being the most common final diagnosis.

**Table 1:** Patients demographic and clinical characteristics.

Variables	Celiac disease	Controls fecal	Controls mucosal
**Number of children**	40	20	19
**Sex (% male sex)**	28%	35%	42%
**Age at presentation in years: median (range)**	10.3 (7.5–15.7)	11.3 (6.8–15.4)	10.6 (2–17.2)
**Breastfeeding (%)**	73%	85%	68%
**Clinical presentation:**			
• Asymptomatic:____	15 (38%)	20 (100%)	0 (0%)
• Anemia:_________	11 (28%)	0 (0%)	1 (5%)
• Diarrhea/A. distention:_	7 (18%)	0 (0%)	0 (0%)
• Growth impairment:___	10 (25%)	0 (0%)	1 (5%)
• Abdominal pain______	10 (25%)	0 (0%)	10 (53%)
• Constipation:_______	8 (20%)	0 (0%)	0 (0%)
• Vomiting:_______	0 (0%)	0 (0%)	3 (16%)
• Dysphagia:________	0 (0%)	0 (0%)	4 (21%)

#### Abundance analysis

**Fig 1:**
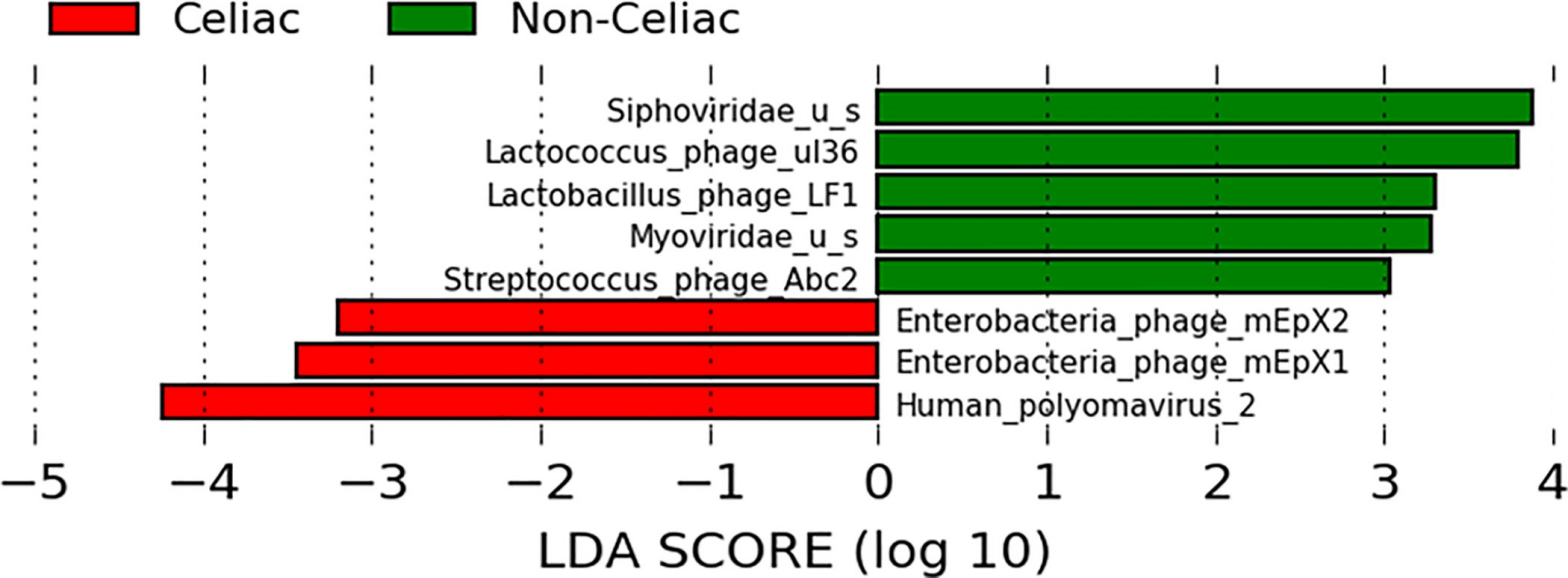
Effect size linear discriminant analysis (LDA) abundance in fecal samples. Bars with positive LDA scores are significantly higher in non-CeD samples (i.e., lower in CeD samples), and bars with negative LDA scores are significantly higher in CeD samples. The figure shows significantly more abundant *Enterobacteria phage mEpX1*, *Enterobacteria phage mEpX1*, and *Human Polyomavirus 2* species in CeD samples and more abundant *Lactococcus phage ul36*, *Lactobacillus_phage Lf1*, and *Streptococcus phage Abc2* species in non-CeD (less abundant in CeD) samples.

There were many more abundant taxa in the fecal than in the mucosal samples of children with CeD. Table 2 highlights significantly more abundant (log2 fold change > 0) and less abundant (log2 fold change < 0) taxa in fecal with corresponding abundance in mucosal samples. The LDA score, depicted in Fig 1, illustrates significant difference in abundance of taxa in stool samples between CeD and non-CeD controls. Significantly more abundant species in children with CeD included *Human polyomavirus 2*, *Enterobacteria phage mEpX1*, and *Enterobacteria phage mEpX2*; whereas more abundant species in non-CeD children (less abundant in children with CeD) included *Lactococcus phages ul36*, *Lactococcus phage LF1*, and *Streptococcus phage Abc2*. In mucosal samples, no taxa were significantly associated with CeD.

**Table 2:** Log2 abundance of viral taxa in fecal compared with mucosal samples.

Level	Organism	Fecal samples		Mucosal samples	
		Log2 abundance	*P- value	Log2 abundance	*P-value
**Family**	Polyomaviridae	7.4160	7.59^−08^	7.2959	0.941
**Genus**	*Betapolyomavirus*	7.7037	3.50^−05^	-0.4227	0.459
**Genus**	*Hp1virus*	0.3228	0.013	0	0
**Genus**	*Begomovirus*	-3.0651	0.025	0	0
**Genus**	*T4virus*	0.7554	0.0252	0	0
**Genus**	*P22virus*	1.7725	0.025	-0.7141	0.197
**Species**	*Enterobacteria phage mEpX2*	3.5008	1.63^−11^	0	0
**Species**	*Enterobacteria phage mEpX1*	5.1193	8.40^−11^	-0.3944	0.497
**Species**	*Escherichia virus Min27*	2.8273	8.36^−09^	0	0
**Species**	*Enterobacteria phage HK630*	1.2555	1.65^−08^	0	0
**Species**	*Escherichia virus Stx2 II*	5.5874	5.86^−08^	0	0
**Species**	*Human polyomavirus 2*	7.7478	1.00^−05^	6.8463	0.987
**Species**	*Enterobacteria phage HK446*	2.4942	< 0.001	0.9879	0.279
**Species**	*Lactobacillus phage KC5a*	0.5627	<0.001	0	0
**Species**	*Lactococcus phage bIL309*	-2.6876	<0.001	0	0
**Species**	*Salmonella phage ST160*	4.4266	<0.001	0	0
**Species**	*Lactobacillus phage phi jlb1*	3.2304	<0.001	0	0
**Species**	*Salmonella phage vB_SemP_Emek*	1.4015	<0.001	0	0
**Species**	*Lactococcus phage ul36*	-2.0110	0.001	0	0
**Species**	*Enterobacteria phage HK542*	1.5753	0.001	0	0
**Species**	*Enterobacteria phage mEp235*	2.2377	0.002	0	0
**Species**	*Salmonella phage SSU5*	-2.2233	0.003	0	0
**Species**	*Enterobacteria phage HK225*	1.2972	0.005	0	0
**Species**	*Vibrio phage pYD38-A*	-1.6063	0.007	0	0
**Species**	*Lactococcus phage r1t*	-3.4189	0.012	0	0
**Species**	*Lactococcus phage bIL312*	-2.0070	0.014	0	0
**Species**	*Watermelon chlorotic stunt virus*	-3.1535	0.014	0	0
**Species**	*Salmonella phage Fels-2*	1.1911	0.015	0	0
**Species**	*Salmonella phage SE1*	-1.4027	0.016	0	0
**Species**	*Lactococcus phage Tuc2009*	-1.4040	0.016	0	0
**Species**	*Enterobacteria phage P88*	3.6610	0.018	-0.1498	0.987
**Species**	*Escherichia virus phiV10*	1.1397	0.019	0	0
**Species**	*Streptococcus phage TP-J34*	0.1024	0.020	0	0
**Species**	*Enterobacteria phage IME10*	2.0204	0.027	-0.5217	0.279
**Species**	*Streptococcus phage Sfi21*	0.2645	0.028	0	0
**Species**	*Shigella phage Sf6*	1.7884	0.030	-0.6420	0.279
**Species**	*Cronobacter phage ENT39118*	0.2255	0.035	0	0
**Species**	*Lactococcus phage bIL310*	-2.2227	0.040	0	0
**Species**	*Enterobacteria phage mEp460*	3.6860	0.041	0.3110	0.623
**Species**	*Lactococcus phage TP901-1*	-1.6033	0.041	0	0
**Species**	*Lactobacillus phage LF1*	-4.8407	0.046	0	0
**Species**	*Streptococcus phage PH15*	2.0586	0.048	0	0

*P-value adjusted for false discovery rate.

#### Breadth and depth analysis

The results are presented in an excel file with three tabs: 1."summarised_data" includes 156 rows, for each of the 39 genomes by each of the four cohorts (mucosa celiac, mucosa non-celiac, stool celiac, stool non-celiac). For each, the metadata and reference information have been provided, along with an average of the coverage depth average, and an average of the coverage breadth, 2."raw_data" includes the mapping information for each sample by each genome. This allows a deeper look at which sample contribute to the values, 3. "pivot_table" was used to aggregate the "raw_data" column into the "summarized_data" tab before the additional metadata columns were added to it (S1 Table).

In addition, a multifasta file shows the sequence of each of the 39 viral species identified with significant differences between CeD and controls (S2 Table).

### Diversity analysis

#### Alpha diversity

**Fig 2:**
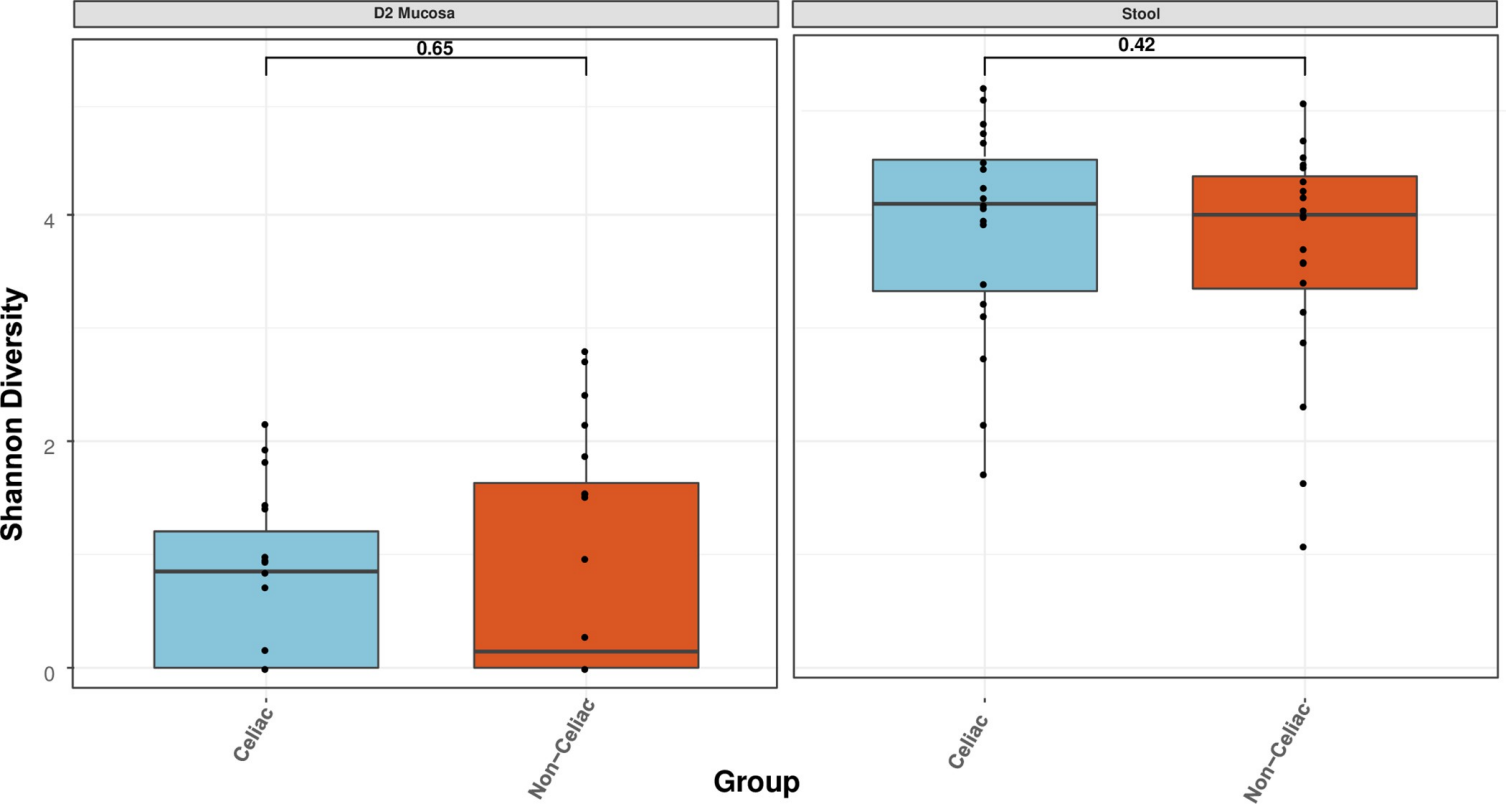
Shannon alpha diversity. Comparison of celiac and nonceliac samples, revealing no statistically significant difference in viral species diversity between mucosal (p = 0.65) or stool (p = 0.42) samples. However, there is a higher alpha diversity in fecal than mucosal samples.

The outcomes of Shannon alpha diversity analysis are presented in Fig 2, highlighting no significant difference existed in diversity between CeD and non-CeD mucosal (P = 0.65) or stool (P = 0.42) samples. However, there was a higher diversity in stool than mucosal samples in CeD samples as well as controls.

#### Beta diversity

**Fig 3:**
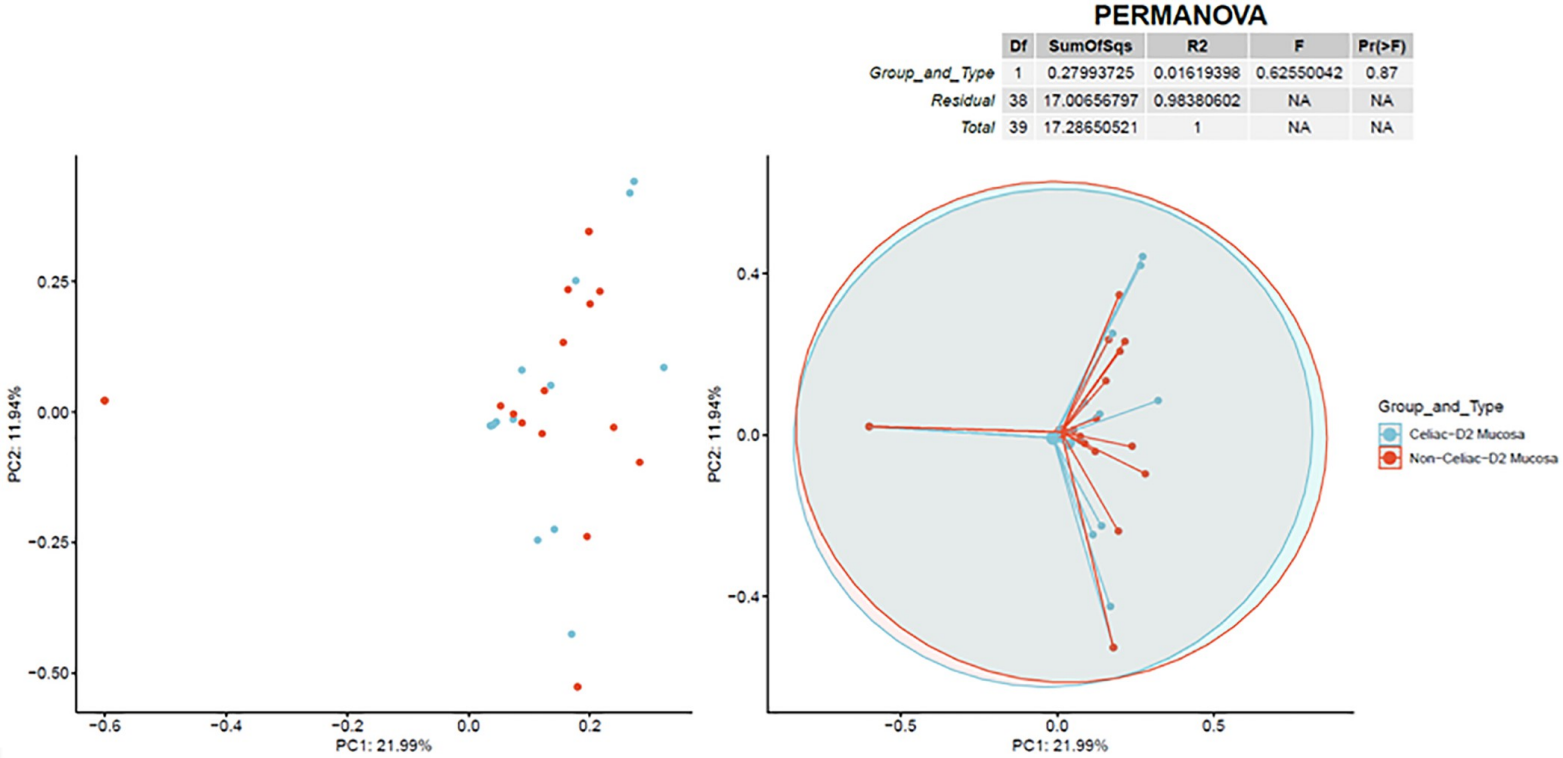
Bray-Curtis beta diversity in mucosa. Two-dimensional principal coordinate analysis for viral species in mucosal samples showing almost complete overlap of CeD samples celiac (CeD) and non-CeD samples. The right plot overlays a 95% confidence ellipse over each cohort and lines connect all values to the average of the cohort.

**Fig 4:**
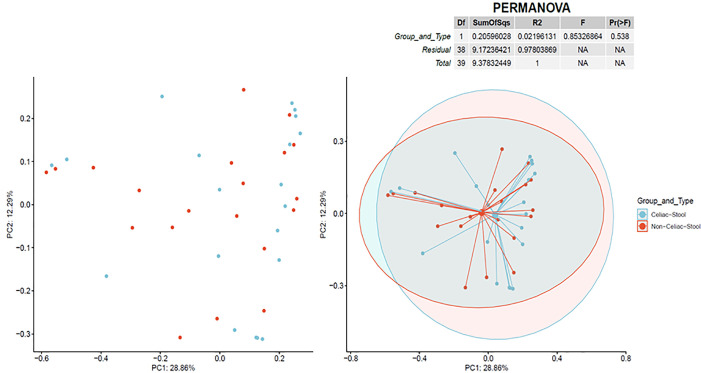
Bray-Curtis beta diversity in stools. Two-dimensional principal coordinate analysis for viral species in stool samples showing almost complete overlap of CeD samples celiac (CeD) and non-CeD samples. The right plot overlays a 95% confidence ellipse over each cohort and lines connect all values to the average of the cohort.

Bray–Curtis diversity analysis results are illustrated in Figs 3 and 4, indicating almost no separation existed between CeD and non-CeD mucosal or stool samples.

## Discussion

The role of viruses in health and disease is well-recognized. Despite the known ability of bacteriophages to infect bacteria and alter the microbiota structure, leading to microbial dysbiosis, studies on intestinal virome are still fragmented, potentially missing an important component of biological networks [36, 37].

In this report, using whole genome analysis, we describe significant associations between viruses and CeD in a cohort of children in the KSA, a country with a high prevalence of CeD (1.5%) [38] as well as a high rate of CeD-predisposing HLA-DQ genotypes (52.7%) [39]. The fact that all children in this study were newly diagnosed and their samples were collected on normal diet containing gluten suggests strong association between the viruses observed and CeD.

To our knowledge, this is the first whole genome description of the virome profile in children with CeD from developing countries who have different culture and lifestyle from Western populations. The finding of significantly more abundant viruses in samples of children with CeD than in controls such as *Human polyomavirus 2*, *Enterobacteria phage mEpX1*, and *Enterobacteria phage mEpX2* suggests a potential “harmful” role. On the other hand, the identification of significantly less abundant viruses such as *Lactococcus phage ul36* suggests a potential “protective” role. Of particular interest is the finding of significantly more abundant *Human polyomavirus 2* (commonly referred to as the JC virus or John Cunningham virus) in children with CeD. This virus, found in normal humans, has been associated with neurologic disease, nephropathy, and cancer [40, 41] but has not been reported, so far, in patients with CeD. Our results contrast with a study by Lindfors, et al. who did not find *Human polyomavirus 2*, *Enterobacteria phage mEpX1*, or *Enterobacteria phage mEpX2* but reported adenoviruses that were not found in our children with CeD [42]. This discrepancy may be explained by differences in the age of children, the methodology, and geographic and population differences between the two studies. Taken together, these findings characterize the presence of viral dysbiosis in children with CeD and support previous reports suggesting a potential role of viruses in general and bacteriophages in particular in children with CeD [43]. Viruses can modify the risk of CeD by interaction with bacteria that affect the immune system. For example, in a study in rats, *Enterobacteria* and *Bifidobacteria* affected the permeability of gliadin-induced intestinal mucosa [44], and in humans, it has been shown that *Bacteroides fragilis*, *E*. *coli*, and *Shigella* could be risk factors that regulate the ability of monocyte recruitment to the mucosa to respond to gliadins and IFN-gamma in CD patients, influencing the course of the disease [45]. Several other possible mechanisms, including the role of some bacterial virulence factors such as microbial transglutaminase as a risk for CeD development, have been extensively reviewed [43]. However, as is true in most microbiome studies, the highly significant association of intestinal virome with CeD in this report does not imply causality.

## Conclusion

The metagenomic analysis of the intestinal virome revealed statistically significant dysbiosis in children with celiac CeD. Further studies with functional analyses to define the relationship of bacteriophages to bacteria and to clarify the role of viruses in CeD might lead to the development of additional treatment options.

## Supporting information

S1 Table Depth and breadth of analysis of viral species. (XLSX)

S2 Table DNA sequences of viral species. (ZIP)
